# Sex Differences in the Performance of 7–12 Year Olds on a Mental Rotation Task and the Relation With Arithmetic Performance

**DOI:** 10.3389/fpsyg.2019.00107

**Published:** 2019-01-30

**Authors:** Marleen van Tetering, Marthe van der Donk, Renate Helena Maria de Groot, Jelle Jolles

**Affiliations:** ^1^Faculty of Behavioral and Movement Sciences, Centre for Brain and Learning, Vrije Universiteit Amsterdam, Amsterdam, Netherlands; ^2^Welten Institute, Research Centre for Learning, Teaching, and Technology, Open University of the Netherlands, Heerlen, Netherlands; ^3^School of Nutrition and Translational Research in Metabolism, Maastricht University, Maastricht, Netherlands

**Keywords:** 3D mental rotation, childhood, early adolescence, mathematics, STEM

## Abstract

This study evaluates boy-girl differences in 3D mental rotation in schoolchildren aged 7–12 years and the relation to arithmetic performance. A dedicated new task was developed: The Mental Rotation Task – Children (MRT-C). This task was applied to a large sample of 729 children. At the age of 7- to 9-years, a sex difference was found in the number of correct judgments made on the MRT-C. Boys performed better than girls. A closer look at the distribution of boys and girls in this age group showed that boys were overrepresented in the top performance quartile, whereas girls were overrepresented in the lowest performance quartile. A second finding was that higher mental rotation performance was significantly correlated to better mathematical achievement. This finding was done for boys, but not for girls. This correlation underscores the important role that spatial processing plays in mathematical achievement and has implications for school practice.

## Introduction

Mental rotation skills play an important role in achievement in Science, Technology, Engineering, and Mathematics (i.e., STEM, see Wai et al., 2009; Wei et al., 2012; Newcombe and Frick, 2010; Bruce and Hawes, 2015). Moreover, previous studies have reported on the importance of 3D mental rotation skills to school geometry performance at the age of 13 years old (Delgado and Prieto, 2004), to mental mathematics at the age of 15–16 years (Kyttälä and Lehto, 2008) and to algebra at the age of 18–25-years (Tolar et al., 2009). Based on these studies, it can be concluded that there are various ways in which 3D mental rotation skills be wielded throughout mathematics in adolescents and (young) adults. It is now of interest to investigate this link in schoolchildren. This is because there is sufficient evidence that spatial reasoning—including mental rotation—is malleable and susceptible to environmental influences, especially in young children (see also Feng et al., 2007; Zelazo and Carlson, 2012; Uttal et al., 2013). If the 3D mental rotation skills of primary school age children are linked to their mathematical achievement, spatial intervention and enrichment programs could be developed to enhance the development of 3D mental rotation skills and thereby facilitate mathematical achievement.

The malleability of spatial skills is also relevant in relation to the well-documented difference between boys and girls in mathematical achievement already at primary school (e.g., Nuttall et al., 2005; Miller and Halpern, 2014; Casey et al., 2015). It may indirectly be the consequence of the preference for spatial play of young boys (Cherney and London, 2006). This preference makes them experienced in spatial skills (Moè, 2016). It gives them a developmental advantage in comparison to girls. The development of spatial abilities – including 3D mental rotation skills – of girls could thus be lagging behind just because they have fewer experiences with spatial play. If these sex differences in spatial skills contribute to differences in the mathematical achievement of boys and girls, intervention programs that stimulate the development of spatial abilities of young girls are promising. These intervention programs may contribute to reducing the well-documented sex differences in successes and achievements in STEM at later ages (Hango, 2013; Miller and Halpern, 2014). This reasoning motivates our study into the relation between 3D mental rotation ability and mathematical achievement in boys and girls. The purpose of this large-scale study was twofold: first, to investigate the contribution of 3D mental rotation to mathematical achievement in 7–12-year old schoolchildren, and second, to investigate sex differences in these participants. More than 700 children participated in the current study.

A dedicated 3D mental rotation task is needed to investigate 3D mental rotation skills in primary school age children. A typical task to evaluate mental rotation requires the participant to compare series of 3D images of objects. The objects may be identical, but rotated around a vertical or horizontal axis or they may be mirror images of each other (Shepard and Metzler, 1971). The participant is asked to determine as quickly as possible which of the images represent the same object but from another rotation (e.g., Peters et al., 1995; Voyer et al., 1995; Hahn et al., 2010; Titze et al., 2010; Hoyek et al., 2011). Large sex differences on such 3D mental rotation tasks are widely reported in adolescent and adult populations (see Voyer et al., 1995). In primary schoolchildren, however, findings of previous studies have given mixed results.

In reviewing the literature that investigated 3D mental rotation skills in schoolchildren, it is important to note that many different tasks have been used. There are substantial differences between these tasks in their procedures and stimuli. Many researchers in young children have used tasks that offer concrete objects (i.e., figures of animals or airplanes) as to-be-rotated stimuli. For instance, Hahn et al. (2010) investigated sex differences in 5-year-old children using colored drawings of animals. Children were asked to indicate whether drawings were identical or mirror-reversed. They found that boys outperformed girls. Another example is the study of Frick et al. (2013) who studied sex differences in the 3D mental rotation skills of 3–5-year-old children. Children saw pairs of asymmetrical ghost figures in seven orientations. One of the ghosts would fit into a hole if rotated right-side up, while the other ghost was its mirror image and would not fit. A disadvantage of the approaches used by Hahn et al. (2010) and Frick et al. (2013) is that they may prompt children to engage primarily in the recognition of object features; children are able to recognize the same figure as the target figure by comparing the object features of both figures and children are not required to mentally rotate the figures to indicate the correct answer (Hoyek et al., 2011). A second disadvantage of the use of concrete objects as stimuli is that they may elicit an emotional reaction based on the child’s positive or negative experiences with that object. It is well-known that emotional reactions can facilitate or hinder memory processing (Christianson and Loftus, 1990; Bradley et al., 1992). If a concrete object elicits a positive emotional reaction, children may store the image more easily into their memory which can facilitate mental rotation of the image. The same accounts for images that elicit negative emotions: this hinders children to store the image into their memory and it is therefore more difficult to mentally rotate the image. For these two reasons, other kinds of stimuli – such as those of the well-established Vandenberg and Kuse Mental Rotation Task (VMRT) – are better suitable to administer 3D mental rotation skills without confounding by object recognition and emotional factors (Vandenberg and Kuse, 1978; Peters et al., 1995).

**FIGURE 1:**
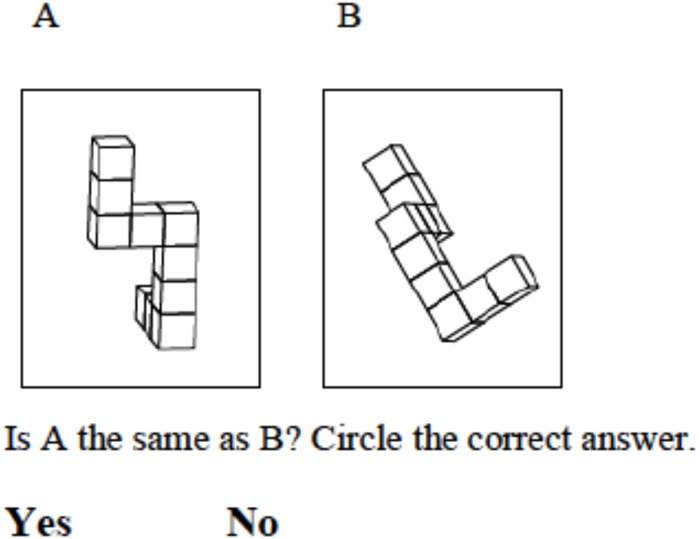
This figure shows an item of the MRT-C. The participant has to mentally rotate the figure on the right to decide whether it matches the target item on the left.

The VMRT has been used by researchers in schoolchildren. For instance, Titze et al. (2010), Hoyek et al. (2011), and Moè (2018) investigate sex differences in 7- to 12-year-old children. The VMRT requires children to mentally rotate three-dimensional cuboid figures (see Figure 1, Shepard and Metzler, 1971). Various researchers concluded that these figures can reliably be used from early ages onwards (see for instance Örnkloo and von Hofsten, 2007 who presented the figures to 22 months old infants, and Moore and Johnson, 2011 who presented the figures to 3 months old infants). The VMRT asks participants to indicate which two out of four cuboid test figures are rotations of the target figure, rather than mirror versions of it. Both Titze et al. (2010) and Hoyek et al. (2011) reported sex differences in children aged 10 years and older, but not in children under the age of 10 (Titze et al., 2010; Hoyek et al., 2011). These researchers therefore concluded that 10 is the age at which sex differences in 3D mental rotation emerge. Moè (2018), on the other hand, reported sex differences from the age of 8 onwards. Previous research findings are thus inconsistent about the age at which sex differences on the VMRT at first emerge.

Close examination of the VMRT as used by earlier studies (i.e., Titze et al., 2010; Hoyek et al., 2011; Moè, 2018) shows why administering it to young children is problematic. The task may not be comprehensible enough to children under the age of 10 because it is a highly complex task, which requires a high working memory capacity. Working memory is required because (1) the participant needs to remember the task instructions and the target stimulus, (2) to mentally rotate the various alternative stimuli one by one and (3) to remember responses to earlier test stimuli whilst mentally rotating the remaining test stimuli. Next to working memory, this task depends upon several other executive functions (e.g., Anderson et al., 2001; Diamond, 2013; Jolles, 2016). Accordingly, (4) planning and prioritizing are necessary. In addition, (5) high levels of selective attention are needed in order not be distracted by other options. Finally (6), the participant needs to suppress the tendency to act before thinking and thus have enough impulse control. It can be concluded that the VMRT is a highly complex task for children and involves various executive functions. Tasks that depend heavily on executive functions can be difficult for 8- to 10-year-old children, and even for many 10- to 14-year-old children. The reason is that executive functions are still immature in childhood, as they continue to develop in childhood until at least early adulthood (Diamond, 2013; Jolles, 2016). If the VMRT is too difficult for children aged less than 10, it is possible that the sex difference in performance goes unnoticed. This notion is substantiated by the finding in the study of Hoyek et al. (2011). These authors found low mean performances amongst 7- to 8-year-old children; the mean number of correct responses was similar for the young boys and girls and they performed equally bad on the task. This floor effect could have masked sex differences. We conclude from this body of research that studying mental rotation in young children requires the use of an age-appropriate task that is not too difficult in order to be sensitive to group differences in performance.

Various researchers have therefore modified the VMRT to use it in schoolchildren. Hawes et al. (2015), for example, reduced task difficulty by using tangible figures as to-be-rotated stimuli, instead of the line drawings of 3D cube figures as in the VMRT. Children aged 4–8 years old needed to indicate which out of three figures was identical to the target figure. The results of Hawes et al. (2015) revealed no sex differences in performance. It is notable, however, that this task is still cognitively demanding for young children because it required a comparison between three possible alternatives. Other researchers have therefore used a binary response approach to reduce cognitive demands (e.g., Heil and Jansen-Osmann, 2008; Hahn et al., 2010; Jansen et al., 2013). For example, Casey et al. (2008), reported sex differences in the performances of 6-year-old children on a 3D mental rotation task with a binary response approach. Children had to indicate whether two tangible cuboid 3D figures were the same or not. Boys outperformed girls on this task. Another example of a study that has used a binary response approach is that of Jansen et al. (2013). They investigated sex differences in 3D mental rotation skills in 8 and 10-year-old schoolchildren. In their study, children had to indicate whether two line drawings of cuboid figures were the same or not. In contrast to Casey et al. (2008), their results revealed no differences between boys and girls in their performances. In fact, they found that the schoolchildren performed beneath chance. Taking the findings of these earlier studies into consideration, it can be concluded that previous studies using modified versions of the VMRT in schoolchildren are inconclusive about the existence of sex differences in 3D mental rotation ability. Our study was therefore carried out to re-investigate the findings of these previous studies. Accordingly, we have modified the VMRT paper-and-pencil test based on the findings of these earlier studies, to make it more suitable for assessing 3D mental rotation in children under the age of 10; the Mental Rotation Task – Children (MRT-C).

In the MRT-C children are asked to indicate whether two stimuli are the same or not. They only have to compare one stimulus with the target, not several, as in the standard VMRT. As our task relies less on executive functions such as working memory, planning and prioritizing and sustained attention, it is easier to apply in young children. In addition to reducing the complexity of the response options, we also reduced the complexity of the stimuli. We limited the stimuli to three-dimensional cuboid figures rotated around a vertical axis by 0 to 180° relative to the target stimulus, whilst the VMRT test stimuli can be rotated around either the horizontal or vertical axis between 0 and 360° (Peters and Battista, 2008). The stimuli for the MRT-C were thus more homogeneous than those for the VMRT and the instructions were easier to understand (Neuburger et al., 2015). This reduced the possibility that children would make procedural mistakes.

In short, there is not enough research to draw conclusions about the age at which the sex gap in 3D mental rotation performance begins to occur (Moè, 2018). Differences between boys and girls in 3D mental rotation skills may contribute to the well-documented sex differences in mathematical achievement that already exists in young children (Miller and Halpern, 2014). They may also contribute to differences between boys and girls in performances and achievement in STEM disciplines at later ages. The aims of the present study were therefore (1) to determine whether there are differences between boys and girls in the performance on the MRT-C in children aged 7–12-years old, and (2) to evaluate the importance of 3D mental rotation ability to mathematical achievement in schoolchildren. We planned a large, cross-sectional study as we wanted to have sufficient power to detect sex differences and to be able to collect information with respect to mathematical achievement at school. Note that previous studies reported an increase in the magnitude of sex differences on 3D mental rotation tasks with age from adolescence onwards (Voyer et al., 1995). It is therefore hypothesized that there are relatively small sex differences in childhood, whereas sex differences in early and later adolescence are more pronounced. A large study sample is needed to detect subtle differences. Our sample therefore consisted of 729 children, and is thereby much larger than that of any previous study (e.g., Voyer et al., 1995; Titze et al., 2010; Hoyek et al., 2011; Jansen et al., 2013). We limited our investigation to children who can be considered to show normal cognitive development; children with evident learning dysfunction and/or problems in the domain of mental health were excluded.

## Materials and Methods

### Participants

The study was part of a large-scale cross-sectional research program called BrainSquare (in Dutch: BreinPlein), which took place in the period of January to June 2016. BrainSquare was aimed at improving knowledge about child-related determinants of learning performance and neurocognitive development of children and young adolescents aged 7 to 12 years (i.e., grades 2 to 6). A total of 1,081 participants were recruited from nine mainstream primary schools in a rural area in the greater Amsterdam region of the Netherlands. Schools were part of the same board and provided roughly equivalent numbers of children from low, middle and high socio-economic status (SES) families. This was done to homogenize our sample with respect to SES. Accordingly, the nine schools were matched on their SES. The SES of the school was established using a composite score that was calculated based on the mean educational levels, incomes, and positions on the labor market of all habitants in the neighborhood of the school in 2016 (Status Scores, 2016). The SES of the schools gives a suitable approximation of the SES of the family in which children grow up in the Netherlands (Central Office for Statistics, 2016). As the study sample included roughly equivalent numbers of children from low, middle and high SES, it is prevented that SES differences between children influenced our main outcomes.

In total, *N* = 1,081 children participated in the study. Participants were excluded based on the following criteria: (a) skipping or repeating a class (*n* = 231), (b) missing data about the participants age (*n* = 46) or sex (*n* = 4), (c) missing data on the mental rotation task (*n* = 54), and (d) unreliable data because the child did not understand the task-instructions (*n* = 17). By excluding the participants that skipped or repeated a grade, we homogenized the sample by including only the typically developing participants in each grade. All children in the sample can be considered healthy, and the sample is a representative selection of normal and healthy children in primary school. The final sample consisted out of *n* = 729 individuals (48.8% girls). Of these participants, 137 subjects were in grade 2 (50.4% girls; Mage = 7.75, *SE* = 0.02), 123 participants were in grade 3 (41.5% girls; Mage = 8.82, *SE* = 0.03), 156 participants were in grade 4 (52.6% girls; Mage = 9.84, *SE* = 0.02), 132 participants were in grade 5 (47.7% girls, *M*age = 10.76, *SE* = 0.03), and 181 participants were in grade 6 (50.3% girls, *M*age = 11.88, *SE* = 0.03). An analysis of variance (ANOVA) revealed that the average age of boys and girls in each grade did not significantly differ between the sexes (*p*-values between 0.28 and 0.96).

For statistical analyses in which sex differences were investigated, the participants were analyzed in two age groups: one group consisting of 416 participants with a mean age of 8.9 years (grades 2–4; 46.2% girls; age range = 7.3–10.4, *SE* = 0.05) and one group consisting of 313 participants with a mean age of 11.4 years (grades 5 and 6; 50.3% girls; age range = 10.3–12.9, *SE* = 0.04). Again, ANOVA revealed that the average age of girls and boys did not significantly differ in the younger age group [*F*(1,414) = 0.06, *p* = 0.81, η_p_ = 0.00], and in the older age group [*F*(1,311) = 0.16, *p* = 0.69, η_p_ = 0.00].

To evaluate the importance of mental rotation to mathematical achievement, all children were included with complete data on a standardized mathematical achievement test. This included 121 (49.6% girls) participants in grade 2, 108 (39.8% girls) participants in grade 3, 129 (50.4% girls) participants in grade 4, 110 (45.5% girls) participants in grade 5 and 121 (51.2% girls) participants in grade 6.

### Procedure

First, the collaborating schools agreed to include the testing procedure into their regular school schedule. Then, parents or caregivers (referred to as caregivers in the rest of the paper) of the participating schools received an information letter about the study and gave written informed consent. Children gave verbal consent to participate. Participation was voluntary. All caregivers were informed that no personalized data would be used in the analyses and that no personalized results would be obtained, since all data were assembled on group level. The Ethical Committee of the Faculty of Behavioural and Movement Sciences of the Vrije Universiteit Amsterdam approved the study protocol.

The children were tested at their own school during normal class time. Questionnaires and neuropsychological tests were administered by means of group administration. This was procedurally identical for every class. A maximum of 30 children was tested together in the classroom. Administration of the total protocol took approximately 60 min. All schools were tested within 3 weeks. Tests were administered by the same two neuropsychologists. One of them gave instructions to the participants and kept track of time. The other walked around in the classroom to assist the school teacher with procedural problems. Additionally, the teacher supported with task administration and kept order in the class.

The data analyzed in the study are part of a larger study protocol consisting of eight neuropsychological tests. Participants first filled in their sex, handedness and their date of birth. The mental rotation task was the sixth task within this protocol and took about 5 min to administer. After task administration, data on the mathematical achievement of each individual child were provided by the school.

### Measures

#### The Mental Rotation Task – Children

Participants had to solve the Mental Rotation Task – Children (MRT-C), which is a newly made, modified version of the VMRT. The VMRT is a well-established and frequently used task to administer mental rotation ability (Vandenberg and Kuse, 1978; Peters et al., 1995). Both the VMRT and the MRT-C have a similar experimental approach. They are both paper-and-pencil tests that use the 10-block, three-dimensional cuboid figures (i.e., originally introduced by Shepard and Metzler, 1971).

The MRT-C consists of 26 items of three-dimensional-objects, with one reference figure on the left and one figure on the right (see Figure 1). All items are derived from the original VMRT and have therefore proven to be valid to assess mental rotation ability (Vandenberg and Kuse, 1978). The total test was divided into two sets, each containing 13 items. Only figures with rotations in space ranging from 0 to 180° around the vertical axis were selected. The participants had to mentally rotate the target figure and indicate whether the figure on the right matched the reference figure. Earlier studies have proven that this approach (two-answer approach) can validly be used in our age-group (Heil and Jansen-Osmann, 2008; Hahn et al., 2010; Jansen et al., 2013). Participants thus needed to answer a question with binary answer approach: yes or no. All items had a similar difficulty level. They were semi-randomly distributed over the two trails based on their rotation. Furthermore, it was prevented that items did or did not match the target item more than three times in a row to control for answer tendencies (individuals answer yes because this was the answer three times in a row). Finally, split-half reliability was checked for each set and revealed that the first half of the set was as difficult as the second half of the set.

The task-instructions of the MRT-C were explained classically by the researchers using an example item. Then, participants were instructed to solve another item themselves. The answers given by the participants were checked for their accuracy by the researchers. It was then asked whether the task-instructions were completely understood. Each set consisted of five pages. Three items were presented on one page in a booklet (sized 210 by 297 mm). The last page of each set contained only one item. Participants were allowed 2 min to complete each set; a short pause of approximately 1 min was given in between. This pause was devised to reduce possible mental fatigue effects. All participants received the same items in the same order. Credit was given for each item that was correctly marked within the 2 min. Total score for an individual participant could thus range from 0 to 26. Also, the number of mistakes was counted for each individual.

The test had a good split-half reliability (Pearson correlation = 0.60, *p* < 0.01). Only 0.7% of the children received a score of 2 on the test (this was the lowest score obtained), and 0.5% of the children received a score of 26 out of a possible 26 (no participants in grade 2, 1 participant in grade 3, 1 participant in grade 4, no participants in grade 5 and 3 participants in grade 6). These findings indicate that there were no floor or ceiling effects.

#### Mathematical Achievement: The Cito Test

Mathematical achievement was assessed with a nationally used paper-and-pencil achievement test, which is standardized and norm-referenced in the Netherlands. This test has been developed by the Dutch Standard Central Institute for Test Development [i.e., in Dutch: Centraal Instituut voor Toetsontwikkeling (Janssen et al., 2010)]. The Dutch Cito mathematics test was used to assess mathematical abilities (Janssen et al., 2010). Participants fill out their answers on a piece of paper. The test took 40–45 min to administer. In grades 3 to 6, the following math skills are covered in the test: (a) number and number relations; (b) addition and subtraction; (c) multiplication and division; (d) measuring (e.g., weights, length, surface, time). From grade 4, (e) percentages and fractions are also covered.

The internal consistency of the Cito mathematics test as a measure of reliability is reported to be high (i.e., for grades 3–6 it ranges from 0.91 to 0.97, see Janssen et al., 2010). The validity of the Cito mathematics test is considered to be high as well since (1) calibration research showed that the differences in participant performance could be explained by one unidimensional concept, (2) similar abilities that were measured with other subparts of the Cito mathematics test were highly correlated, and (3) participants’ performances on the Cito mathematics test was predictive for performance on the following Cito test.

In the present study, the “skill-scores” (i.e., translated from the Dutch “vaardigheidscores”) was used as a measure for cognitive performance. These scores are known to improve over the years and are useful in monitoring the progression on each Cito test (Janssen et al., 2010). There are two different test moments for each grade, one regularly administered halfway through the year (January) and one around June. In this study, we used the Cito test results obtained in January 2016.

#### Statistical Analyses

All analyses were performed using SPSS version 23. Eta squares were reported as a measure for effect sizes. A total of seven analyses were performed. At first, Pearson correlations were calculated between the Cito mathematical achievement and MRT-C performance in each grade. Secondly, Pearson correlations were calculated for boys and girls separately in each grade. Thirdly, it was investigated whether boys and girls differed in their mathematical achievement per grade using separate one-way analyses of variance (ANOVAs). Modified Hochberg correction was used to control for multiple testing issues; a *p*-value of <0.01 was considered statistically significant.

Fourthly, two (age group: younger aged 7- to 9-years old vs. older participants aged 10- to 12-years old) x two (sex: boys vs. girls) ANOVAs were performed with MRT-C performance (total number of correctly identified items) as dependent variable. A *p*-value of <0.05 was considered statistically significant. Because of the significant interaction between grade and sex, *post hoc* one-way ANOVAs were performed to investigate sex differences in each age group separately. Modified Hochberg correction was used to control for multiple testing issues when assessing sex differences in the two separate age groups. According to this correction, a *p*-value of ≤0.04 was considered critical for assigning statistical significance (Rom, 2013). Then, to investigate more precisely at what age possible sex differences emerge, *post hoc* one-way ANOVAs were performed to investigate sex differences in each study grade. Again, Modified Hochberg correction was used to control for multiple testing issues; a *p*-value of <0.01 was considered critical for assigning statistical significance (Rom, 2013).

The fifth analyses were performed to take a closer look at the distribution of boys and girls in the overall sample. MRT-C performance was divided into quartiles ranging from lowest to highest performances (according to a procedure published in Dekker et al., 2013). This was done per grade to control for the age effect which was needed, because MRT-C performance was expected to improve with grade. These analyses provide more insight into the distribution of boys and girls in a group of low, medium, good, and excellent performers. This reflects a real-life situation, since each class includes performers of various levels.

The sixth analyses were performed using one-way ANOVAs to investigate whether boys and girls (independent variable) differed in their total number of mistakes on the MRT-C (dependent variable) per age group. These analyses were performed to control for the possibility that boys performed better because they prioritized speed above accuracy and thereby achieved a higher number of correct responses because they have been guessing the solutions to some items. According to the Modified Hochberg correction that was used to control for multiple testing issues; a *p*-value of ≤0.04 was considered critical for assigning statistical significance (Rom, 2013).

## Results

### Correlations Between Mathematical Achievement and MRT-C Performance

In the total study population, MRT-C performance was significantly correlated to performance on the mathematical achievement test in grades 2–5 (see Table 1).

**Table 1:** Pearson correlations between MRT-C performance and mathematical achievement.

	*N* (boys/girls)	*r* Total	*r* Boys	*r* Girls
Grade 2	121 (61/60)	0.38^∗∗^	0.27^∗^	0.39^∗∗^
Grade 3	108 (65/43)	0.23^∗^	0.26^∗^	0.16
Grade 4	129 (64/65)	0.19^∗^	0.13	0.15
Grade 5	110 (60/50)	0.26^∗^	0.31^∗^	0.17
Grade 6	121 (59/62)	0.16	0.117	0.22

*^∗^*p* < 0.05, ^∗∗^*p* < 0.01.*

The correlation was then investigated for boys and girls separately. Results revealed that MRT-C performance was significantly correlated to mathematical achievement of boys in grades 2–5. For girls, MRT-C performance was significantly correlated to mathematics achievement in grade 2.

### Sex Differences in Mathematical Achievements

Differences in the mathematical achievement between boys and girls were investigated per grade. Results of one-way ANOVAs revealed significant differences in the mean mathematical achievement between boys and girls in grade 2 [*F*(1,119) = 8.76, *p* < 0.01, η^2^ = 0.07)] and grade 4 [*F*(1,127) = 9.35, *p* = 0.03, η2 = 0.07)]. Mean performances of boys (grade 2: *M* = 175.2, *SD* = 29.6; grade 4: *M* = 90.5, *SD* = 10.9) were higher than that of girls (grade 2: *M* = 159.8, *SD* = 27.5; grade 4: *M* = 83.9, *SD* = 13.6). Mean difference in mathematical achievement between boys and girls approaches significance in grade 5 [*F*(1,108) = 4.46, *p* = 0.04, η^2^ = 0.04)] (see Table 2). Mean mathematical achievement of boys (*M* = 105.4, *SD* = 11.2) was higher than that of girls (*M* = 100.3, *SD* = 13.9).

**Table 2:** Differences between boys and girls in mathematical achievement per grade.

	Boys	Girls		
	Mean (SD)	Mean (SD)	η^2^	*p*-Value
Grade 2	175.2 (29.6)	159.8 (27.5)	0.07	<0.01*
Grade 3	154.2 (76.3)	141.4 (71.4)	0.01	0.38
Grade 4	90.5 (10.9)	83.9 (13.6)	0.07	<0.01*
Grade 5	105.4 (11.2)	100.3 (13.9)	0.04	<0.04*
Grade 6	114.7 (9.9)	115.5 (11.3)	<0.01	0.69

*^∗^*p* < 0.05.*

### Sex Differences in Younger and Older Participants in Mental Rotation

Differences between boys and girls, younger and older children, and the possible interaction between sex and age group on MRT-C performance were investigated. Table 3 presents the number of correct substitutions on the MRT-C by age group and sex. Results revealed significant main effects of sex [*F*(1,725) = 14.80, *p* < 0.01, η_p_ = 0.02] and age group [*F*(1,725) = 92.28, *p* < 0.01, η_p_ = 0.11] on MRT-C performance. Boys (*M* = 16.6, *SE* = 0.26) showed better performance than girls (*M* = 15.2, *SE* = 0.26), and the older participants (*M* = 17.8, *SE* = 0.26) showed better performance than the younger participants (*M* = 14.5, *SE* = 0.23). The interaction between sex and age-group on MRT-C performance was significant as well [*F*(1,725) = 5.22, *p* = 0.02, η_p_ = 0.01], indicating that the difference in the performance of boys and girls is different in the older age group than in the younger age group.

**Table 3:** Mean performance on the MRT-C for younger and older participants, and for boys and girls.

	Total sample	Boys	Girls		
	M (SE)	M (SE)	M (SE)	η_p_	*p*-Value
Total sample	15.9 (0.19)	16.6 (0.26)	15.2 (0.26)	0.02	0.01^∗^
Younger	14.5 (0.23)	15.5 (0.33)	13.4 (0.31)	0.05	<0.01^∗^
Older	17.8 (0.26)	18.1 (0.40)	17.5 (0.34)	0.00	0.30

*^∗^*p* ≤ 0.01.*

Because of the significant interaction, *post hoc* analyses were performed to investigate sex differences within each age group. Results showed an effect of sex on MRT-C performance in the younger age group [*F*(1,414) = 22.01, *p* < 0.01, η_p_ = 0.05], but not in the older age group [*F*(1,311) = 1.06, *p* = 0.30, η_p_ = 0.00]. More specific, younger boys (*M* = 15.5, *SE* = 0.33) outperformed younger girls (*M* = 13.4, *SE* = 0.31), whereas in the older age group boys and girls performed equally.

#### *Post hoc* Analyses: Sex Differences per Grade

*Post hoc* analyses were conducted in which sex differences on MRT-C performances were investigated in each grade separately. Results showed an effect of sex on MRT-C performance in grade 2 [*F*(1,135) = 9.15, *p* < 0.01, η_p_ = 0.06] and in grade 4 [*F*(1,154) = 11.82, *p* < 0.01, η_p_ = 0.07], to the advantage of boys. The sex-difference on MRT-C performance approached significance in grade 3 [*F*(1,122) = 4.61, *p* = 0.03, η_p_ = 0.03], to the advantage of boys. No sex differences were found in grade 5 [*F*(1,130) = 0.77, *p* = 0.38, η_p_ = 0.01], and 6 [*F*(1,179) = 0.44, *p* = 0.51, η_p_ = 0.00]. Means, standard errors, *p*-values and effect sizes are presented in Table 4.

**Table 4:** Mean performance on the MRT-C and results of the analyses for boys and girls per grade.

	Boys	Girls
	M (SE)	M (SE)	η_p_	*p*-Values
Grade 2	13.5 (0.50)	11.4 (0.52)	0.06	<0.01^∗^
Grade 3	15.8 (0.60)	13.8 (0.66)	0.03	<0.03
Grade 4	17.1 (0.53)	14.8 (0.41)	0.07	<0.01^∗^
Grade 5	17.6 (0.61)	16.8 (0.58)	0.01	0.38
Grade 6	18.4 (0.53)	18.0 (0.41)	0.00	0.51

*^∗^*p*-Value ≤ 0.05.*

### Distribution of Boys and Girls in the Total Study Population

**FIGURE 2:**
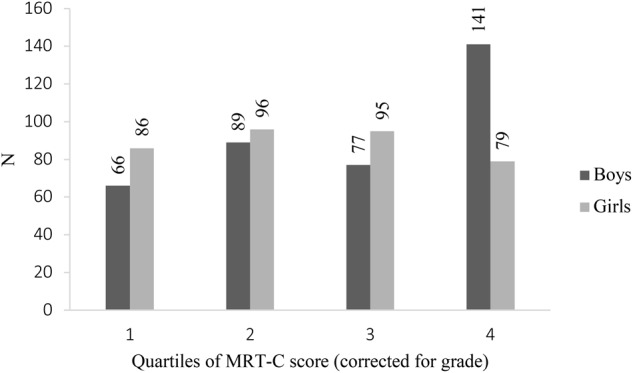
Sex differences in MRT-C performance in the total population divided over quartiles. Quartile 1 = 25% lowest MRT-C scores: quartile 4 = 25% highest MRT-C scores. ^∗^*p*-Value ≤ 0.01.

Analyses were performed to take a closer look at the distribution of boys and girls in the overall sample. The distribution of boys and girls differed significantly between the quartiles [χ^2^(3) = 21.87, *p* < 0.01]. It appeared that students in the highest quartile were predominantly boys (boy: girl ratio = 2: 1; boys: *z* = 2.7). There were no significant differences in the boy: girl ratio in the first (boy: girl ratio = 3:4; boys: *z* = -1.3), second (boy: girl ratio = 1: 1; boys: *z* = -0.6) and third quartiles (boy: girl ratio = 3: 4; boys: *z* = -1.2) (see Figure 2).

#### Distribution Boys and Girls in the Younger and Older Age Groups

The distribution of boys and girls was investigated in the younger and older age group. Within the younger age group, we found that the relative number of boys and girls significantly differed between the quartiles [χ^2^(3) = 19.052; *p* < 0.01]. It appears that students within the lowest quartile were predominantly girls (boy: girl ratio = 2:3; boys: *z* = -2.8), and within the highest quartile were predominantly boys (boy: girl ratio = 7: 3; boys: *z* = 2.4). There were no significant differences in the boy: girl ratio for the second (boy: girl ratio = 1: 1; boys: *z* = -1.0) and third (boy: girl ratio = 1: 1; boys: *z* = -0.8) quartiles.

Within the older age group, we found that the differences in the distribution of boys and girls approached significance [χ^2^(3) = 7.159; *p* = 0.067]. It appeared that students within the third quartile were predominantly girls (boy: girl ratio = 2: 3; boys: *z* = -2.1), and within the highest quartile were predominantly boys (boy: girl ratio = 3: 2; boys: *z* = 2.3). There were no significant differences in the boy: girl ratio for the first (boy: girl ratio = 1: 1; boys: *z* = -0.2) and second (boy: girl ratio = 1: 1; boys: *z* = -0.3) quartiles.

### Sex Differences in the Number of Mistakes

Additional analyses were performed to investigate whether boys and girls differed in their total number of mistakes. Results showed that within the younger group, girls (*M* = 7.1, *SE* = 0.29) made significantly more mistakes than boys (*M* = 6.0, *SE* = 0.34), *F*(1,414) = 6.10, *p* = 0.01, η_p_ = 0.02). In the older group, no significant difference in the total number of mistakes was found between boys (*M* = 4.8, *SE* = 0.31) and girls (*M* = 5.2, *SE* = 0.29), *F*(1,311) = 0.69, *p* = 0.41, η_p_ = 0.02.

## Discussion

This aims of this study were (1) to investigate the correlation between 3D mental rotation and mathematical achievements in 7–12-year-old children, and (2) to investigate whether sex differences in 3D mental rotation were present before the age of 10 years. The MRT-C has been developed for investigating 3D mental rotation performance in children below the age of 10 years. Results revealed that MRT-C performance was positively correlated to higher mathematical achievement, especially for boys. Moreover, there were differences between boys and girls in their mathematical achievements. Boys performed better than girls. The same was found with respect to sex differences on the MRT-C; boys performed better than girls. This sex difference was confined to the younger age group (aged 7–10 years old). Major strength of our study was its large sample size which enabled us to detect this relatively small, but substantial difference in contrast to earlier studies that were much smaller and therefore unable to detect this difference (for instance, Hoyek et al., 2011 investigated sex differences in 22 and 66 participants, and Titze et al., 2010 investigated sex differences in 95 participants). The importance of this finding was substantiated by inspection of the sex distribution of performance in the younger group. Boys were overrepresented in the top performance quartile, whilst the lowest quartile was predominantly made up of girls. The same was found in the older group: there were more boys than girls in the top performance quartile. Finally, analyses showed that girls made more mistakes than their male peers at the ages of 7- to 10-years, but not at the ages of 10- to 12-years. These analyses were performed to control for the possibility that boys performed better because they prioritized speed above accuracy and achieved a higher number of correct responses because they guessed the answers to some items. This alternative hypothesis was not supported by the data as boys made fewer mistakes than girls. These findings substantiate the notion that boys are better in the task than girls at the age of 7–10 years.

The important finding that 3D mental rotation performance was positively correlated to mathematical achievements in 7–11-year-olds is in line with that of earlier studies in older participants. These studies showed that 3D mental rotation skills are involved in various aspects of mathematics. For instance, 3D mental rotation skills are involved in school geometry when visualizing the lengths of lines or the size of in-depth-figures (Delgado and Prieto, 2004). They are also involved in mental mathematics while holding multiple simultaneous representations of numbers into mind (Kyttälä and Lehto, 2008; Thompson et al., 2013). Our finding that young boys are better in 3D mental rotation than girls the same age indicates that boys could be better in visualizing and thinking about representations of numbers into their minds than girls at early ages. This could be beneficial for their mathematical achievements because our results showed that mental rotation performance was significantly correlated to mathematical performance especially in boys. Our finding is substantiated by that of Frick (2018). She reported better mental transformation skills, particularly the ones requiring a high level of spatial flexibility and a stronger sense for spatial magnitudes in boys than in girls. She also found that these skills were beneficial for mathematical performance. This is an important finding when it comes to improving the mathematical achievements of girls. It could explain why boys generally outperform girls in mathematical related fields, as has extensively been reported (e.g., Miller and Halpern, 2014). An interesting implication of our findings is that children should be stimulated to get experience in spatial information processing (mental rotation and other spatial skills) as this can aid in the development of skills that are important for mathematical thinking. It can be envisaged that this applies not only to boys but also to girls; our finding suggest that mathematical achievements of girls could be improved by practicing their spatial abilities. This suggestion should be evaluated in controlled intervention experiment. Moreover, for future research it would be relevant to investigate whether alternative strategy use could be a source of sex differences on MRT-C performance in this age-group. In an adult population, for instance, Boone and Hegarty (2017) found that males outperformed females when items were structurally different so that mental rotation was not necessary. They also found that when all foils were structure foils and participants were instructed to look for structure foils, the significant sex difference was no longer evident. Their findings therefore indicate that there are sex differences in strategy use (males look for structure foils and females do not) that contribute to the sex difference in mental rotation performance. It would now be interesting to investigate whether this sex difference already exists in young children. This would indicate that mental rotation performance of girls could improve if they learn more efficient strategies.

With respect to the task used, our findings support the notion that the original VMRT is too complex for young children. The task could therefore be insensitive to sex differences. This hypothesis is supported by the fact that the adjusted VMRT (i.e., the MRT-C) is less complex in both the answer approach and the nature of the stimuli used. In contrast to studies using the VMRT (Titze et al., 2010; Hoyek et al., 2011; Hawes et al., 2015), our study did reveal sex differences in 7–10-year-old children. This finding is in line with that of Casey et al. (2008), who used the same answer approach in their task as that of the MRT-C (i.e., their approach was also binary). Because of this simplified answer approach, MRT-C performance is less dependent on executive functions such as working memory, planning and prioritizing and selective attention than performance on the VMRT. It appears that this is important for research in young children given the existence of individual differences in executive functions in 7–12-year-old children (e.g., Anderson et al., 2001; Diamond, 2013; van Tetering and Jolles, 2017). For instance, there is evidence that the child’s sex is a relevant factor contributing to individual differences in executive functions (see van Tetering and Jolles, 2017). When difficult tasks – such as the VMRT – are used to assess 3D mental rotation, sex differences in executive functions may interfere with task performances. This unwanted contamination of performance on the target skill can be avoided by using a more straightforward task such as the newly developed MRT-C.

An important strength of the MRT-C is the use of three-dimensional cuboid figures. These stimuli are unlikely to elicit emotional reactions that could influence mental rotation performance. This is one of the reasons that these figures have been used in many earlier studies on sex differences in 3D mental rotation ability in primary school age children (e.g., see Voyer et al., 1995; Titze et al., 2010; Hoyek et al., 2011). Another reason why previous studies used these figures is that there is much evidence that participants actually mentally rotate stimuli of this type into an upright position in order to determine whether pairs of stimuli are identical or mirror images (Hoyek et al., 2011). Moreover, Hawes et al. (2015) concluded that cuboid figures can be used by 4-year-old children. They showed that these children performed above chance in their task using these figures. Cuboid figures are thus highly appropriate stimuli to administer mental rotation ability, and they are useful in young children.

An additionally relevant finding of this study pertains to the fact that sex differences in 3D mental rotation are present in the best performing older children: boys are overrepresented in the upper performance quartile, whereas there was no sex difference in MRT-C on a group level in early adolescents aged 10- to 12-years old. This finding implies that sex differences are especially present in the extreme performance groups including children with excellent 3D mental rotation skills. For instance, if sex differences are investigated in the total study population, substantial sex differences in the extreme performance groups are canceled out by the smaller sex differences in the average performance groups. This may explain why Jansen et al. (2013) did not find differences between 10-year-old boys and girls on their 3D mental rotation task. Our finding highlights the importance for future research to investigate sex differences in the extreme performance groups.

Furthermore, we did not find a sex difference on MRT-C performance on a group level in the older age group. This suggests that the task is too easy for use with older children (adolescents above 10 years of age). When a task is too easy, performance is subject to a ceiling effect. That is, all groups perform nearly perfectly within the time they have to perform the task, and so there is no scope for detection of group differences. This notion is substantiated by the fact that 44% of the youngest children belonged to the top 33% highest performers, whereas 67% of the oldest age group belonged to the 33% highest performers. Our finding that the mean performance of boys and girls is similar in the older group is also substantiated by additional analyses. These show that older boys and girls make an equivalent number of mistakes. This is an important finding and implies that age-appropriate tasks should be used when assessing cognitive abilities such as 3D mental rotation. New investigations of potential sex differences in MRT-C performance in children aged 9 years and older should be performed with a more difficult version of the task. The task difficulty can easily be increased by expanding the range of possible rotations (e.g., to between 0 and 360° around the vertical or horizontal axis, as in the VMRT; Neuburger et al., 2015).”

### Practical Implications

This study has a practical implication with regard to the stimulation of mental rotation skills and related spatial activities in children who lag behind in this function, notably young girls. It is known that spatial activities – such as spatial navigation and experiences in spatial play – could stimulate the maturation of brain networks underlying mental rotation ability (e.g., Krendl et al., 2008; Haier et al., 2009; Jaušovec and Jaušovec, 2012; Jolles and Crone, 2012; Dunst et al., 2013; Lowrie et al., 2017). Upon the structural changes in the brain, also the function of the brain areas involved improve (Jolles and Crone, 2012). For instance, Hawes et al. (2013) and Fernández-Méndez et al. (2018) showed that young children learn mental rotation as a result of carefully designed activities and lessons targeting the cognitive skill. Also, Nazareth et al. (2013) showed that the significant relation between the sex of the participant and MRT score is partially mediated by the number of masculine spatial activities participants had engaged in during their youth. Performing spatial activities thus both improves brain maturation and mental rotation skills. On this basis, it can be hypothesized that boys and girls develop similar mental rotation abilities when they are equally exposed to relevant spatial activities and encouraged to perform such activities (Nazareth et al., 2013).

There are various activities that involve spatial cognition, such as those involving the engagement of the total body while navigating throughout the environment. Other activities require more subtle motor movements, such as when building a tower out of wooden blocks. These kinds of activities require mental rotation of the wooden blocks and the to-be-build tower (Jansen and Heil, 2010). The importance of such activities to school achievements has recently been provided by Giles et al. (2018). They showed that young children with better eye-to-hand coordination were more likely to achieve higher scores for reading, writing, and math. Teachers and caregivers should therefore encourage girls to engage in a variety of such spatial activities inside and outside of school. This is important because of the importance of spatial abilities for many daily life activities, such as finding one’s way in three-dimensional space (e.g., to go to school, sports and playing games, see Newcombe and Frick, 2010). In addition, children’s mental rotation abilities are fundamental to quantitative reasoning, such as in mathematics and geometrics, which requires the use of spatial cues, making comparisons and mentally visualizing, rotating and calculating the sides two- and three-dimensional figures (Nuttall et al., 2005; Rosselli et al., 2009; Jirout and Newcombe, 2015). Improving girls’ mental rotation performance may lead to later success and achievement in the domain of STEM.

## Conclusion

This study showed that 3D mental rotation ability was positively correlated to mathematical achievement in 7–12-year-old children. We also showed that sex differences in 3D mental rotation emerge at least at the age of 7 years, to the advantage of boys. These findings are important with respect to improving sex differences in mathematical achievements and in STEM related disciplines. They suggest that interventions that stimulate the development of spatial skills may facilitate mathematical achievements, especially of young girls. Based on our results, we conclude that MRT-C is suitable for young children. Nevertheless, our results highlight the need to use age-appropriate tasks when assessing cognitive abilities, as we did not find sex differences in mean performance of children aged 10- to 12-years old. Future research is needed to fine-tune the MRT-C to make it suitable to both younger and older children.

## Author Contributions

MvT gave substantial contributions to the conception and design of the work, analysis and interpretation of the data for the work, drafting of the work and final approval of the version to be published, and finally gave agreement to be accountable for all aspects of the work in ensuring that questions related to the accuracy or integrity of any part of the work are appropriately investigated and resolved. MvdD and RdG gave substantial contributions to the design of the work, interpretation of the data for the work, critical revision of the work for important intellectual content and final approval of the version to be published, and finally agreement to be accountable for all aspects of the work in ensuring that questions related to the accuracy or integrity of any part of the work are appropriately investigated and resolved. JJ gave substantial contributions to the conception or design of the work, acquisition and interpretation of data for the work, drafting of the work, critical revision of the work for important intellectual content and final approval of the version to be published, and finally agreement to be accountable for all aspects of the work in ensuring that questions related to the accuracy or integrity of any part of the work are appropriately investigated and resolved.

## Conflict of Interest Statement

The authors declare that the research was conducted in the absence of any commercial or financial relationships that could be construed as a potential conflict of interest.
